# Seizures Caused by Exposure to Hyperbaric Oxygen in Rats Can Be Predicted by Early Changes in Electrodermal Activity

**DOI:** 10.3389/fphys.2021.767386

**Published:** 2022-01-05

**Authors:** Hugo F. Posada-Quintero, Carol S. Landon, Nicole M. Stavitzski, Jay B. Dean, Ki H. Chon

**Affiliations:** ^1^Department of Biomedical Engineering, University of Connecticut, Storrs, CT, United States; ^2^Department of Molecular Pharmacology and Physiology, Morsani College of Medicine, University of South Florida, Tampa, FL, United States

**Keywords:** electrodermal activity, seizures, central nervous system oxygen toxicity, Sprague Dawley rats, HBO_2_

## Abstract

Hyperbaric oxygen (HBO_2_) is breathed during undersea operations and in hyperbaric medicine. However, breathing HBO_2_ by divers and patients increases the risk of central nervous system oxygen toxicity (CNS-OT), which ultimately manifests as sympathetic stimulation producing tachycardia and hypertension, hyperventilation, and ultimately generalized seizures and cardiogenic pulmonary edema. In this study, we have tested the hypothesis that changes in electrodermal activity (EDA), a measure of sympathetic nervous system activation, precedes seizures in rats breathing 5 atmospheres absolute (ATA) HBO_2_. Radio telemetry and a rodent tether apparatus were adapted for use inside a sealed hyperbaric chamber. The tethered rat was free to move inside a ventilated animal chamber that was flushed with air or 100% O_2_. The animal chamber and hyperbaric chamber (air) were pressurized in parallel at ~1 atmosphere/min. EDA activity was recorded simultaneously with cortical electroencephalogram (EEG) activity, core body temperature, and ambient pressure. We have captured the dynamics of EDA using time-varying spectral analysis of raw EDA (TVSymp), previously developed as a tool for sympathetic tone assessment in humans, adjusted to detect the dynamic changes of EDA in rats that occur prior to onset of CNS-OT seizures. The results show that a significant increase in the amplitude of TVSymp values derived from EDA recordings occurs on average (±SD) 1.9 ± 1.6 min before HBO_2_-induced seizures. These results, if corroborated in humans, support the use of changes in TVSymp activity as an early “physio-marker” of impending and potentially fatal seizures in divers and patients.

## Introduction

Extended exposure to hyperbaric pressure while diving followed by decompression can lead to gas bubble formation. While all gases produce bubbles upon decompression, nitrogen is problematic because it is inert and comprises 78% of air. Thus, excessive production and accumulation of nitrogen bubbles causes decompression sickness (DCS; Curly et al., 1997; Weathersby et al., 1999; Schipke and Pelzer, 2001; Chouchou et al., 2009; Bai, 2011; Gempp et al., 2012; Gempp and Louge, 2013). Breathing hyperbaric oxygen (HBO_2_) is an effective treatment for DCS because recompression diminishes the size of the gas bubbles. Furthermore, the ensuing large partial pressure gradient for nitrogen diffusion from tissues-to-blood-to alveolar gas promotes denitrogenation; that is, removal of nitrogen from the body through pulmonary ventilation (Moon, 2014). Likewise, prior to decompression, prebreathing 100% oxygen – oxygen prebreathe – is an effective method to mitigate DCS in certain situations (Lund et al., 2000; Blogg et al., 2003; Dainer et al., 2007). Finally, HBO_2_ is also used during military and recreational diving operations and, additionally, during HBO_2_ therapy for treating problematic wounds (Ciarlone et al., 2019).

What limits the use of HBO_2_ in these situations is the increased risk of developing central nervous system oxygen toxicity (CNS-OT). CNS-OT is often preceded by a host of early non-convulsive signs and symptoms (S/Sx) characterized by enhanced autonomic activity and abnormal cardiorespiratory changes that ultimately culminate in generalized tonic–clonic seizures and loss of consciousness followed by cardiogenic pulmonary edema (Donald, 1992; Arieli et al., 2006; Ciarlone et al., 2019). Losing consciousness and convulsing under water could result in the dislodgement of the diver’s gas supply from his or her mouth, and likely lead to drowning (Donald, 1947; Natoli and Vann, 1996; Arieli et al., 2006; Clark and Neuman, 2008). Likewise, losing consciousness and convulsing in patients undergoing HBO_2_ therapy can result in further injuries (Hampson and Atik, 2003). Developing tools that measure the early physiological changes that precede seizures are useful for predicting the onset of seizures due to CNS-OT; for example, paradoxical hyperoxic hyperpnea or hyperventilation during extended exposure to HBO_2_ is a physiological marker (“physio-marker”) of impending seizures in mammals (Dean et al., 2004; Pilla et al., 2013b).

The safe latent period for breathing HBO_2_ without seizures is variable within and between individuals, and the risk for developing seizures in humans increases during protracted breathing of >2.5–3.0 atmospheres absolute (ATA) O_2_. Rodents, likewise, breathing 3 ATA O_2_ inside a dry, pressurized hyperbaric chamber, also develop generalized seizures, but it requires from 3 to 5 h and results in onset of pulmonary oxygen toxicity (Simon and Torbati, 1982; Demchenko et al., 2007). For this reason, studies using unanesthetized animals employ higher levels of HBO_2_, ranging from 4–5 ATA O_2_ (Demchenko et al., 2000; Ciarlone et al., 2019). The higher level of HBO_2_ accelerates onset of CNS-OT without developing the confounding problems of pulmonary oxygen toxicity (Dean et al., 2003; Ciarlone et al., 2019).

Physiologically, elevated sympathetic activity associated with augmented seizure activity occurs at 1 ATA when breathing air (Wannamaker, 1985). Presumably, increased autonomic activity that precedes seizure genesis in mammals breathing HBO_2_ will reveal a similar pattern of increased electrodermal activity (EDA; Ciarlone et al., 2019). Therefore, a sensitive measure of early changes in sympathetic neural activity would be a suitable tool – a “physio-marker” – for seizure detection if it occurs.

EDA has been used increasingly to assess the level of sympathetic activity (Freeman and Chapleau, 2013). The EDA results from the changes in conductance in the skin (Boucsein et al., 2012). Remarkably, sudomotor activity is known to be solely controlled by the sympathetic nervous system, making the EDA a purely sympathetic assay (Dawson et al., 2007; Boucsein et al., 2012). In humans, EDA is linked to central sympathetic activity as demonstrated by the significant surge in EDA amplitude preceding epileptic seizures (Poh et al., 2010, 2012). In rodents, good EDA signals can be obtained from subdermal electrodes placed on a rat’s back (Kita et al., 1975; Hata et al., 1981). And while rats have limited sweating only at the paws, changes are EDA in rats have been reported that correlate with stress (Kita et al., 1975; Hata et al., 1981).

Human’s EDA signal (microsiemens, μS) is typically analyzed in time domain (Boucsein et al., 2012). The slow modulations of EDA are called the tonic component, whereas the faster modulations of EDA are termed the phasic component. The phasic component includes the skin conductance responses (SCRs), which are those rapid transient events observed in the EDA signal. There is no available tool for tonic/phasic decomposition of EDA in rats. Nevertheless, to better capture the dynamics of EDA linked to sympathetic tone, we previously developed a time-varying analysis method for EDA (Posada-Quintero et al., 2016b). Our quantitative method, termed TVSymp, has shown to be more sensitive in determining stress-induced changes than the widely used methods in the literature (Posada-Quintero et al., 2016b, 2017, 2020a). TVSymp can be easily adjusted for rats’ EDA by exploring the specific spectral content of the signal.

We have also proven that the sensitivity of EDA to sympathetic arousal is maintained under the water (Posada-Quintero et al., 2018a). This suggests that EDA could be used to track the early manifestations of CNS-OT under the water. Since the TVSymp has shown to be highly sensitive and consistent in humans, the goal of this study was to test the hypothesis that prior to seizure onset there is a significant increase in the amplitude of the phasic component of EDA captured by TVSymp in rats. If this holds and the results are subsequently validated in humans, it can be used as a “physio-marker” for prediction of CNS-OT seizures in humans based on TVSymp.

In this study, we aimed to develop methods to measure EDA in freely behaving rat and determine the feasibility of using changes in EDA for predicting the onset of seizures resulting from CNS-OT caused by prolonged breathing of HBO_2_. We hypothesize that changes in EDA can be used to predict seizures in rats breathing HBO_2_ if O_2_-induced seizures are preceded by a similar significant surge in EDA as previously observed before epileptic seizures (Poh et al., 2010, 2012). To test this hypothesis, adult male Sprague Dawley rats were implanted with subdermal needle electrodes and exposed to 5 atmospheres absolute (ATA) HBO_2_ in a sealed hyperbaric chamber. Seizures were detected using behavioral and neurological criteria though video recording and telemetric recordings of cortical electroencephalogram (EEG) activity. This animal model of CNS-OT has been successfully used for measuring changes in behavior, heart rate and minute ventilation that precede seizures and onset of CNS-OT (Pilla et al., 2013b).

## Materials and Methods

### Animals

All experiments used male, Sprague Dawley rats (*n* = 10, 338 ± 43 g, $\bar{x}$ ± SD) purchased from Envigo Laboratory and maintained on a 12-h light:dark cycle. All experiments were performed in accordance with animal care and use protocols approved by the University of South Florida Institutional Animal Care and Use Committee (PHS assurance no. A4100-01; AAALACi 434) and the Department of the Navy, Bureau of Medicine and Surgery.

### Surgeries: Telemetry Module and Subdermal Electrodes

Seven of ten animals underwent two surgeries. The first survival surgery was to implant a radio telemetry transmitter module, either the DSI-4ET or -F40 module (Data Sciences International, St. Paul, Minnesota), for recording cortical EEG activity from the motor cortex in an awake, freely behaving rat. The second surgery was less invasive and was done at least one week later, on the day of the experimental dive. The other 3 animals used in the study only received the subdermal needle electrodes on the day of the experiment. Animals were anesthetized initially with 3–5% isoflurane (in O_2_) and then maintained on 2.5% isoflurane (in O_2_) for the duration of surgery (30–60 min depending on which telemetry module was implanted). Carprofen was given preoperatively, then 5–6 h after the end of surgery, and finally 12 and 24 h after surgery (5 mg/kg of bw, sc). The surgical site on the animal was prepared for sterile surgery using standard practices; DSI-4ET modules were implanted as described by Pilla et al. (2013a,b) and DSI-F40 modules were implanted as reported in Held et al. (2014).

**Figure 1:**
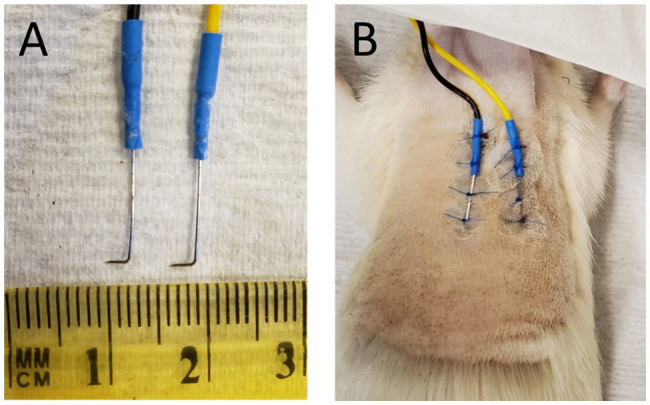
**(A)** Subdermal needle electrodes after removal from animal, postmortem. The length of the tip distal to the 90^°^ bend ranged from 1 to 3 mm. (**B)** Subdermal needles implanted in the back of a euthanatized animal prior to removal.

A pair of parallel subcutaneous needle electrodes (NeuroGuard S46-637, 14 mm × 0.38 mm, stainless steel; Consolidated Neuro Supply, Inc., Milford, Ohio) were implanted in all ten animals. Each 1.5-meter long, insulated electrode lead was shortened to 27 inches and the factory attached connector discarded. A fine gold pin (Fine Science Tools, No. 19003-00) was soldered to the exposed wire and reinforced with heat shrink tubing. Each electrode needle tip was bent to a 90° angle, 1 to 3 mm from the tip, using a pair of micro-needle nose pliers (Figure 1A). Electrical continuity between the bent electrode and pin was confirmed using an Ohm meter.

The animal was anesthetized using isoflurane as described above. Fur on the animal’s back torso was removed using electric clippers, exposing an area measuring approximately 3 × 3 inches. Exposed skin was then cleaned in the following order with chlorohexidine gluconate (Hibiclens) antispectic/antimicrobial solution, PVP iodine, and sterile saline. Needle electrode tips were sterilized in a hot bead “Germinator” and then attached to the back or the torso using non-absorbable suture (Figure 1B). The tips of the subdermal electrodes were positioned to point laterally and angled down into the skin but not too deep. Typically, insertion of the tips to a maximum depth of <1-mm to a minimum depth of dimpling and barely penetrating the dermis gave the best results. Placing the electrode tips too deeply beneath the surface of the skin produced a large DC offset that could not be adjusted during “subject zero” of the software (see below) thereby preventing measurement of electrodermal resistance. Likewise, initial attempts using 8 mm diameter Ag-AgCl disc electrodes (MLA0118, ADInstruments) mounted to exposed skin with conductive gel, and glued in place, failed to provide reliable measurements of EDA. Once the needle electrodes were sutured in place, Vetbond surgical glue was applied at the suture knots.

### Tethering the Rat With Implanted Subdermal Electrode Leads

All tethering components were purchased from Lomir Biomedical Inc. (Malone, NY). The rat tether (RT-12D, 12-inches long, sewn) was shortened from 12 to 9 inches. While the animal was still breathing isoflurane through the nosecone and anesthetized, the pair of subdermal electrode leads (27-inches long) were passed through the Velcro end of the tether (nearest rat) and out the opposite end. A rat jacket (RJ 01) of appropriate size was fitted over the rat’s front limbs and drawn together over the needle electrodes on the animal’s back, securing the Velcro fittings of the jacket to the tether, and stabilized in place using thick suture to lace the jacket’s eyelets together and close the jacket. The rat was removed from the isoflurane nosecone and transferred with its jacket-tether assembly to the equipment sled extended out from the open hyperbaric chamber. Before connecting the needle leads to the pigtail lead attached to the electrical panel inside the hyperbaric chamber, an “open circuit zero” was performed using the LabChart Pro software (version 7.3.2, ADInstruments, Colorado Springs, CO). The subdermal electrode leads were then connected to the pigtail leads (Fine Science Tools, No. 19003-01) to complete the circuit and the “subject zero” was performed for LabChart Pro. Subject zeroing was performed while holding the anesthetized rat perfectly still. The distal end of the animal’s needle leads was disconnected from the internal electrical panel pigtail and the rat then transferred back to the lab bench and 2.5% isoflurane anesthesia continued by nosecone.

The tether containing the two electrode leads, now secured to the jacket and extending from the back of the anesthetized animal, was fed upwards through an opening in the inverted wire lid of the animal cage (11.0 × 7.25 × 5.0 inches) and attached to swivel assembly mounted on the cage lid. The swivel assembly, which permitted unrestrained movement once the rat aroused, consisted of the rat swivel-tether connector block (RST4, double swivel to double tether (with Allen key)), rat swivel (RS PF1H, plastic with hallow center), and swivel retainer (RS 07 “shoebox” for double). The inverted wire lid was secured to the animal cage by passing zip ties through predrilled holes in the animal cage.

### Animal Exposure Chamber and Main Hyperbaric Chamber Setup

**Figure 2:**
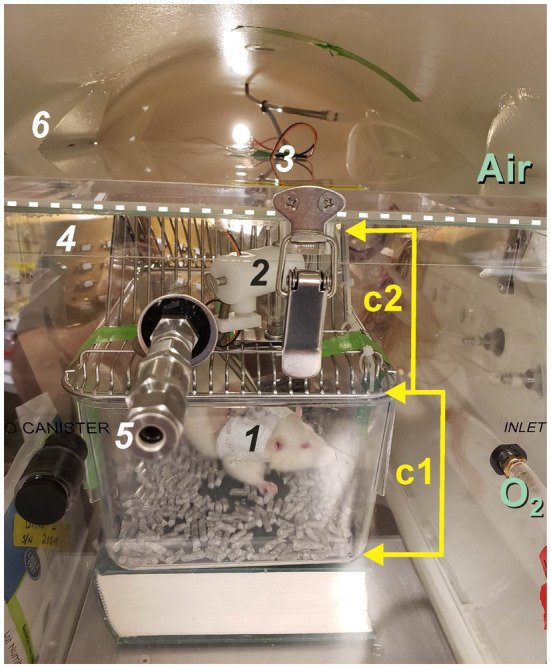
The animal exposure chamber inside the hyperbaric chamber. The main hyperbaric chamber houses the animal exposure chamber, which contains the animal cage (c1) and wire cage lid (c2). The main chamber is compressed using air only while the animal exposure chamber is ventilated with either air at 1 ATA (control) or 100% O_2_ at 1 or 5 ATA (hyperoxia). In this experiment, the front edge of the lid to the animal exposure chamber is demarcated by the horizontal white dashed line; one of six buckles used to seal the cover of the animal exposure chamber is seen here. A preset pressure relief valve (5) prevents over-pressurization of the animal exposure chamber during the experiment. Other important items seen here include the animal’s jacket and tether (1) that passes up through an opening in the wire cage lid (c2) and connects to the moveable tether assembly (2) mounted on the cage lid (c2). The two, needle electrode leads pass upwards through the tether cable to the tether assembly (2), exit through the roof the animal exposure chamber (3), and plug into the internal electrical connector panel behind the animal exposure chamber (4). The welded window port (6) is barely visible in this image through which the animal’s behavior is monitored continuously using a video camera. This animal was not implanted with a radio telemetry module; however, if it was the receiver would have been stood on its side and resting against the exterior of the animal exposure chamber. Cabling for the telemetry receiver then plugs into the internal electrical connector panel (4).

**Figure 3:**
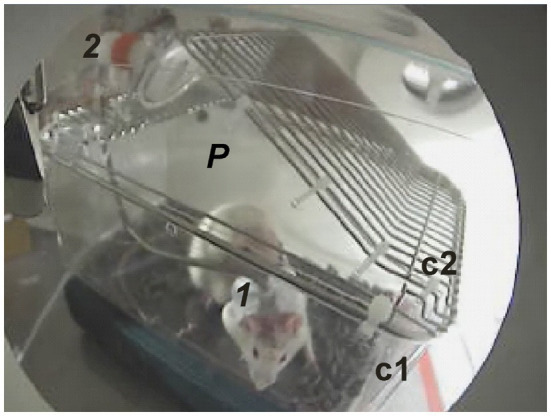
A video image captured at the beginning of an experiment showing the rat’s jacket and tether (1) and the tether assembly (2) mounted on the inverted wire cage lid (c2). The metal triangular-shaped side panel was removed, which normally reinforces the depression that supports an animal’s water bottle and replaced with a plexiglass panel (P) so the animal inside the cage (c1) could be viewed using the video camera.

The hyperbaric system setup consisted of two chambers that were compressed/decompressed in parallel. The main hyperbaric chamber (Reimers Systems model 20–48 duo- hyper−/hypobaric research chamber; PCCI Hyperbaric Systems, Alexandria, VA) was ventilated with air (FO_2_ = 0.21) and when sealed has an internal dimension of 20 × 49 inches, an internal volume of ~205 liters, and a maximum working pressure of 7.8 ATA (100 psig); see Figure 2 in Pilla et al. (2013b) and Figure 1B in D’Agostino et al. (2013). It also contained the radio telemetry receiver and electrical connector panel (Figures 2, 3). The animal exposure chamber was the second main chamber and was made from a veterinary anesthesia induction chamber (cats, 19.75 × 12.0 × 12.0 inches; model V102-V, RWD Life Science, Inc., San Diego, CA). The front of the animal exposure chamber is visible in Figure 2 and its preset pressure release port (5). The animal exposure chamber contained the rat cage (Figure 2, c1 & c2) and tether assembly (1 & 2). The experiments reported here all used compressed and dried air generated from an oil-less rotary scroll compressor (model ES04 ECOscroll, F.S. Curtis) and dryer (SMC, model IDFB4E-11 N; BCH Mechanical, Largo, FL).

The top lid of the animal exposure chamber is removable and seals in place with six latches. Gas ports at opposite ends provided continuous ventilation of the rat with either air (control) or 100% oxygen at 1 ATA and during compression to 5 ATA O_2_. The two inlet and outlet ports that penetrate the hyperbaric chamber and connect to input and output ports of the animal exposure chamber are visible in Figure 2 at the 3 o’clock position. The inlet port to the animal exposure chamber is visible toward the lower right of Figure 2. (O_2_). The subdermal needle electrode leads (3) pass through a small hole predrilled in the lid for attaching the handle, which was removed and discarded. The needle electrode leads connect to the pigtail plugged into the internal electrical panel (4). The metal siding on one side of the water bottle support on the lid was cut away using a Dremel tool and replaced with a triangular piece of plexiglass (Figure 3, *P*). This way the animal in the holding cage (c1 & c2) could be viewed unobstructed through the window port of the hyperbaric chamber using the video camera once the animal cage and tether assembly were placed inside the animal exposure chamber and slid inside the hyperbaric chamber (Figure 3).

The animal exposure chamber is not a pressure rated hyperbaric chamber; however, because the main hyperbaric chamber and animal exposure chamber are ventilated in parallel, with only a 1–3 psig differential between them, the animal exposure chamber can be safely compressed to 5 ATA O_2_. A preset pressure release valve (+8 psig) prevents over-pressurization of the animal exposure chamber. The animal exposure chamber was maintained ~1–3 psig higher than the hyperbaric chamber in case a leak develops thus preventing hyperbaric chamber air from leaking into the animal exposure chamber and diluting the 100% O_2_ atmosphere the rat was breathing.

### Dive Profile

Once the rat was removed from anesthesia and installed in the tether harness and rat cage it usually awoke within 5 min. The experiment did not begin, however, until the rat had been off isoflurane at least 90 min and breathing 1 ATA air. After 90 min of recovery, the experiment began by collecting 15-min of baseline of control data in 1 ATA air. This was followed by 15-min of data breathing 1 ATA O_2_. Thus, each rat was off isoflurane at least 2 h before commencing the dive. The animal was then compressed at ~1 atmosphere (~15 psig) per minute to maximum “depth,” 5 ATA O_2_ (60 psig; 132 feet of seawater). The experimental dive lasted until onset of neurological seizures (↑ cortical EEG activity) and/or visible seizures (video recording) or until a maximum exposure time of 90-min if no seizures occurred. Gas exiting the animal exposure chamber was sampled continuously for %O_2_ and %CO_2_ to ensure the animal was breathing either air (21% O_2_) or 100% O_2_ and that metabolic CO_2_-rebreathing did not occur (<1% CO_2_; Gemini O_2_-CO_2_ monitor, CWE, Inc.). Adiabatic warming of the atmosphere inside the main hyperbaric chamber during compression, and cooling during decompression, were quickly dissipated through the steel walls of the pressure vessel. This resulted in a modest elevation in air temperature inside the animal chamber during compression to 5ATA, which stabilized at ~1 to 1.5°C above control, predive air temperature (Pilla et al., 2013b).

### Measuring EDA and Chamber Pressure

Subdermal needle electrode signals passed from the internal connector panel through the wall of the hyperbaric chamber via a high-pressure electrical penetration and then terminated in an external electrical connector panel and ultimately the FE116 GSR amplifier (galvanic skin response amplifier, fully isolated AC excitation, and automatic zeroing low-voltage amplifier, 22 mVrms at 75 Hz) and PowerLab data acquisition system (LabChart Pro, version 7.3.2), which digitized EDA data at 100 Hz, with 12 bits resolution (ADInstruments, Inc.). Hyperbaric chamber pressure and air temperature data were also collected using LabChart Pro. In addition, EDA, chamber pressure and air temperature were collected using the P3 Ponemah Physiology Platform (version 5.20), which was configured to acquire and record telemetry signals (cortical EEG activity, body temperature), non-telemetry signals (via the ACQ 7700, including hyperbaric chamber pressure and air temperature, and EDA), and a continuous video signal from each experiment (D’Agostino et al., 2013; Pilla et al., 2013b; Held et al., 2014). The telemetry receiver was maintained inside the hyperbaric chamber and rested on its side against the exterior of the animal exposure chamber. The receiver’s telemetric data cable penetrated the hyperbaric chamber wall as described above and elsewhere (Pilla et al., 2013a). In the case of EDA, the input signal from the GSR amplifier to PowerLab (ADInstruments) was shared with the ACQ 7700 (DSI, Inc.).

### EDA Signal Processing

#### Spectral Analysis of EDA in Rats

For the analysis of the spectral content of EDA in rats, we extracted the first two minutes of data from each animal while the chamber was fully compressed. We down-sampled the EDA signals to 4 Hz. In humans, the dynamics of the EDA spectrum are largely confined below 0.15 Hz in resting conditions, 0.25 Hz under stress (postural, physical, and cognitive; Posada-Quintero et al., 2016a), and about 0.37 Hz under vigorous exercise (Posada-Quintero et al., 2018b). Given that there is not information on the spectral distribution of EDA in rats, we have used a sampling frequency high enough to spectrally contain frequencies of autonomic control expectedly higher than in humans (Gehrmann et al., 2000). The power spectra of EDA signals collected in rats were calculated using Welch’s periodogram method with 50% data overlap. A Blackman window (length of 128 points) was applied to each segment, the Fast Fourier Transform was calculated for each windowed segment, and the power spectra of the segments were averaged. Each spectrum was normalized dividing by its total power. We then evaluated the frequencies that contain 95% of the normalized power of the EDA signals for the entire testing sample using the following equation:


$$F_{95\%}=F\;|\left(\frac{\sum_{f=0}^{f=F}\;PSD\left(f\right)}{\sum_{f=0}^{f=2Hz}PSD\left(f\right)}*100=95\right)$$

Where *f* is frequency, *F_95%_* represents the frequencies that contain 95% of the normalized power of the EDA signals, *PSD* is the mean of all power spectral densities of all rats. Note that 2 Hz is Nyquist frequency as the sampling frequency was set to 4 Hz.

#### Preprocessing

**Figure 4:**
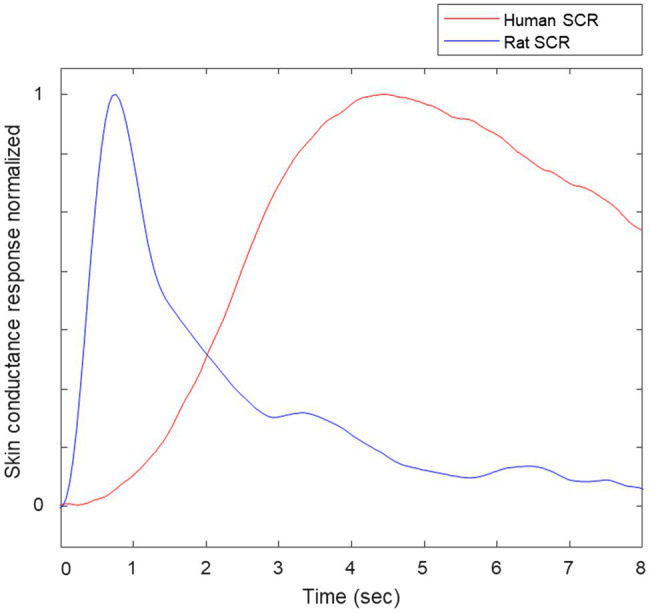
Samples of normalized skin conductance responses (SCRs) of human and rat.

We inspected the signals and videos to prevent any motion artifacts corruption and false positives produced by it. We have adjusted the standard preprocessing procedure for humans’ EDA (Posada-Quintero and Chon, 2020), considering the spectral content of rats’ EDA to be twice as large as humans’ EDA. For illustration purposes, Figure 4 includes samples of skin conductance responses (SCRs) extracted from a human in a different study and a rat from this study, normalized to amplitude = 1. Notice that the rat’s SCR is faster than human’s SCR. We applied a median filter (0.5-s width) and a low-pass finite impulse response filter with cutoff frequency of 2 Hz were applied to remove noise from the selected raw EDA data.

#### Time-Variant Spectral Index of Sympathetic Control Based on EDA, TVSymp

**Figure 5:**
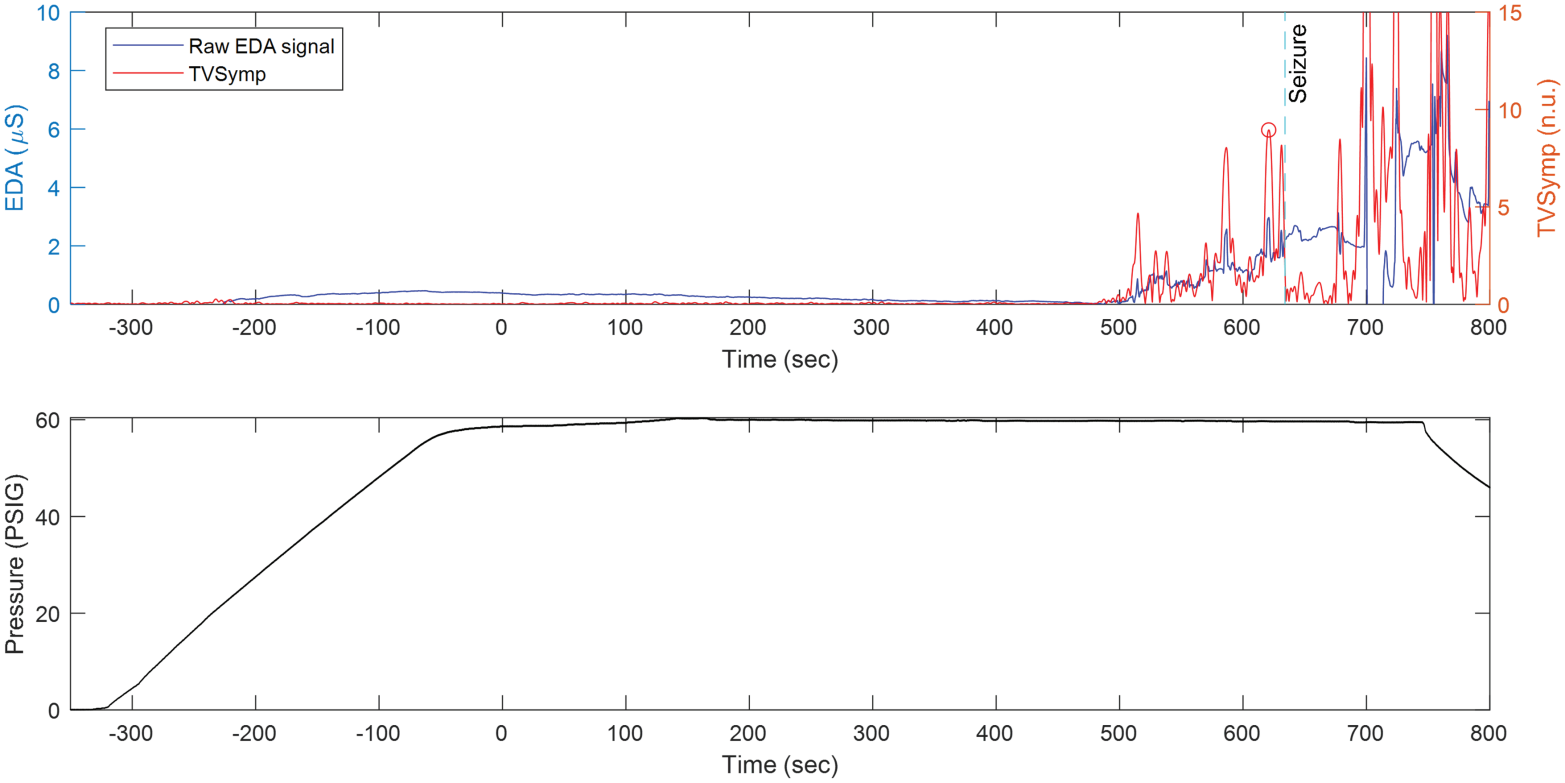
The same animal as in Figure 8 but with the x-axis compressed in time to show continuously measured raw EDA and its derived TVSymp activity before, during, and after seizure. Top: Raw EDA and time-varying spectral analysis of EDA (TVSymp, non-dimensional units) for a rat undergoing exposure to 5 ATA of HBO_2_. Bottom: Profile of the dive.

In humans, we have previously developed the time-invariant spectral analysis of EDA for sympathetic control (TVSymp), to better capture the dynamics of EDA (Posada-Quintero et al., 2016b). We have tested TVSymp for sympathetic tone assessment in applications including stress, fatigue, dehydration, and pain (Posada-Quintero et al., 2016a, 2016b, 2017, 2020a,b). TVSymp has lower intra-subject variability and higher day-to-day consistency and sensitivity, compared to the time-invariant index and the traditional time domain measures of EDA (skin conductance level and the number of skin conductance responses), in response to orthostatic and cognitive stress (Posada-Quintero et al., 2019). To compute the TVSymp, the time-frequency representation of EDA was computed using variable frequency complex demodulation (VFCDM), a time-frequency spectral analysis technique that provides accurate amplitude estimates and one of the highest time-frequency resolutions (Chon et al., 2009). At a sampling frequency of VFCDM decomposition of 4 Hz, the second and third components, comprising the approximate range 0.16–0.48 Hz, were used to compute TVSymp. Amplitudes of the time-varying components in this band are summed together to obtain an estimated reconstructed EDA signal, X’(*t*). X’(*t*) is normalized to unit variance (making it non-dimensional units), and its instantaneous amplitude is computed using the Hilbert transform (Huang et al., 1998). A more detailed explanation of TVSymp can be found in our previous paper (Posada-Quintero et al., 2016b). The resulting instantaneous amplitude corresponds to the TVSymp time series; see below, Figure 5, top panel.

### Statistics

For each rat, we obtained the maximum value of TVSymp within a window of 5 min right after the chamber was pressured (Start) and within a window of 5 min right before the seizure. The normality of the sample of TVSymp values was tested using the one-sample Kolmogorov–Smirnov test (Massey, 1951; Miller, 1956; Wang et al., 2003). All indices were found to be normally distributed. For that reason, we used the t-test (*p* < 0.05) to test the significance of the differences between baseline TVSymp activity during the first two minutes upon reaching 5 ATA O_2_ (time zero) and two-minute intervals preceding seizure for each animal.

## Results

### Measuring EDA in Tethered, Freely Behaving Rats

**Figure 6:**
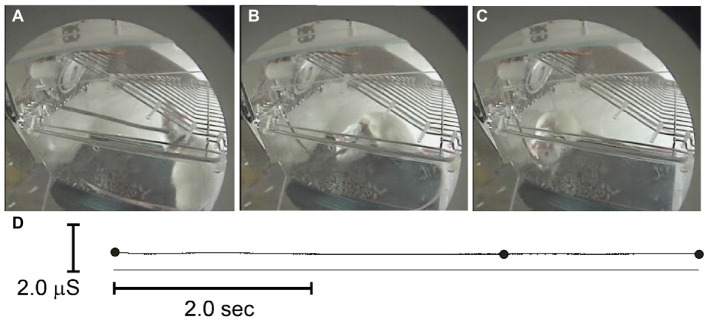
Exploring and walking **(A-C)** in a tethered rat did not affect measured EDA **(D)**. The black dots **(D)** indicate when the video images were sampled in **A–C**.

Figure 6 shows an example of EDA in a rat moving freely about its cage while breathing 1 ATA air at the start of an experiment. The subdermal needle electrode leads are secured in the tether that is visible extending from the animal’s back up to the movable tether assembly at the top left of the cage lid. The cage lid is inverted to give the rat more space to move freely. The elapsed time moving from panel a to c was 8 s. Notice that measured EDA (d) showed no sudden changes in conductivity associated with smooth, moderate movements, such as standing on its rear legs and exploring the cage lid (a) and then dropping down on four legs and walking to the opposite end of the cage (b, c). In general, EDA activity exhibited slow DC fluctuations, on the order of tens of seconds to minutes that did not correlate with either compression or transient thermal fluctuations. Short-lasting, rapid animal movements, however, sometimes caused fast fluctuations in EDA as did onset of intense seizures. Most rats, however, remain calm and quiet during the dive until onset of seizures.

### CNS Oxygen Toxicity Seizures

**Figure 7:**
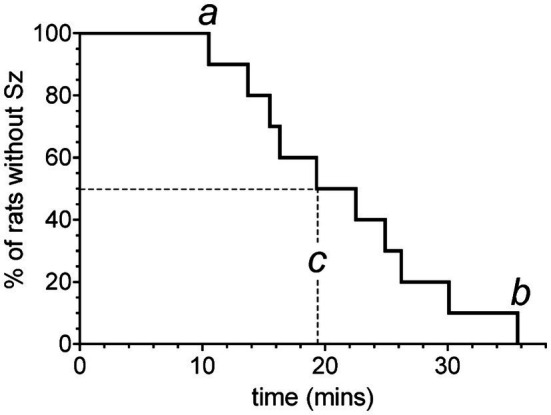
Kaplan–Meier survival curve adapted for reporting the range of latency times prior to onset of seizures while breathing 5 ATA O_2_. Each vertical step indicates the latency time to seizure in a rat after reaching 5 ATA O_2_. Refer to text for definitions of a, b, and c.

Figure 7 shows a Kaplan–Meier survival curve adapted for reporting the range of latency times prior to onset of seizures while breathing 5 ATA O_2_. All animals tested developed seizures after reaching maximum depth and breathing 5 ATA O_2_. Rats seized, on average, at 19.6 min (c), ranging from 10.5 (a) to 35.8 min (b).

### TVSymp Activity Derived From Raw EDA Before and During Seizures at 5 ATA O_2_

**Figure 8:**
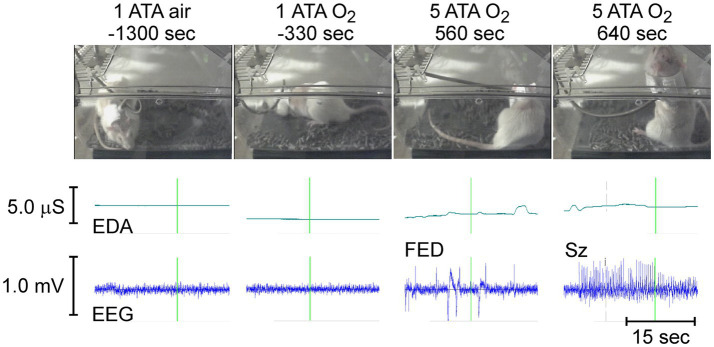
(top to bottom). Animal behavior, EDA, and cortical EEG activity in a rat before (1 ATA air and O_2_) and during exposure to HBO_2_ (5 ATA O_2_). The times demarcated by the vertical green lines in EDA and EEG traces mark the time of the accompanying video image.

Figure 8 shows the behavior, raw EDA, and cortical EEG activity in a freely behaving rat in normobaric air (1 ATA air) and hyperoxia (1 ATA O_2_) approximately 21.5 (−1,300 s) and 5.5 (−330 s) minutes, respectively, before compression to 5 ATA O_2_. Roughly 9 min (560 s) after reaching 5 ATA O_2_ the rat develops increased cortical EEG activity known as first electrical discharge (FED) that precedes seizures in some animals. Oxygen toxicity seizures (Sz) begin slightly more than 1 min later after approximately 10.5 min (640 s) of exposure to 5 ATA O_2_. Sz began as tonic posturing in the supine position (not shown) and evolved to standing posture with recurring clonic seizures of the front limbs (shown) at which point the rat loses balance and falls onto its side while having generalized tonic–clonic seizures (not shown).

Figure 5 shows raw EDA data and derived TVSymp data in the same animal (Figure 8) during exposure to 5 ATA O_2_. Notice that the raw EDA signal (purple trace) exhibited noticeable increases in activity about two minutes before the seizure occurred. In general, the EDA exhibits different dynamics in rats as compared with humans. In humans, the rise time (from the onset to the peak) falls between one and two seconds (Boucsein et al., 2012). Specifically, we have observed that the skin conductance responses (SCRs) in rats are faster, with rise times below one second; see samples of SCRs extracted from a human and a rat (Figure 4). This suggests the spectral content measured in rats’ EDA is composed of higher frequencies.

**Figure 9:**
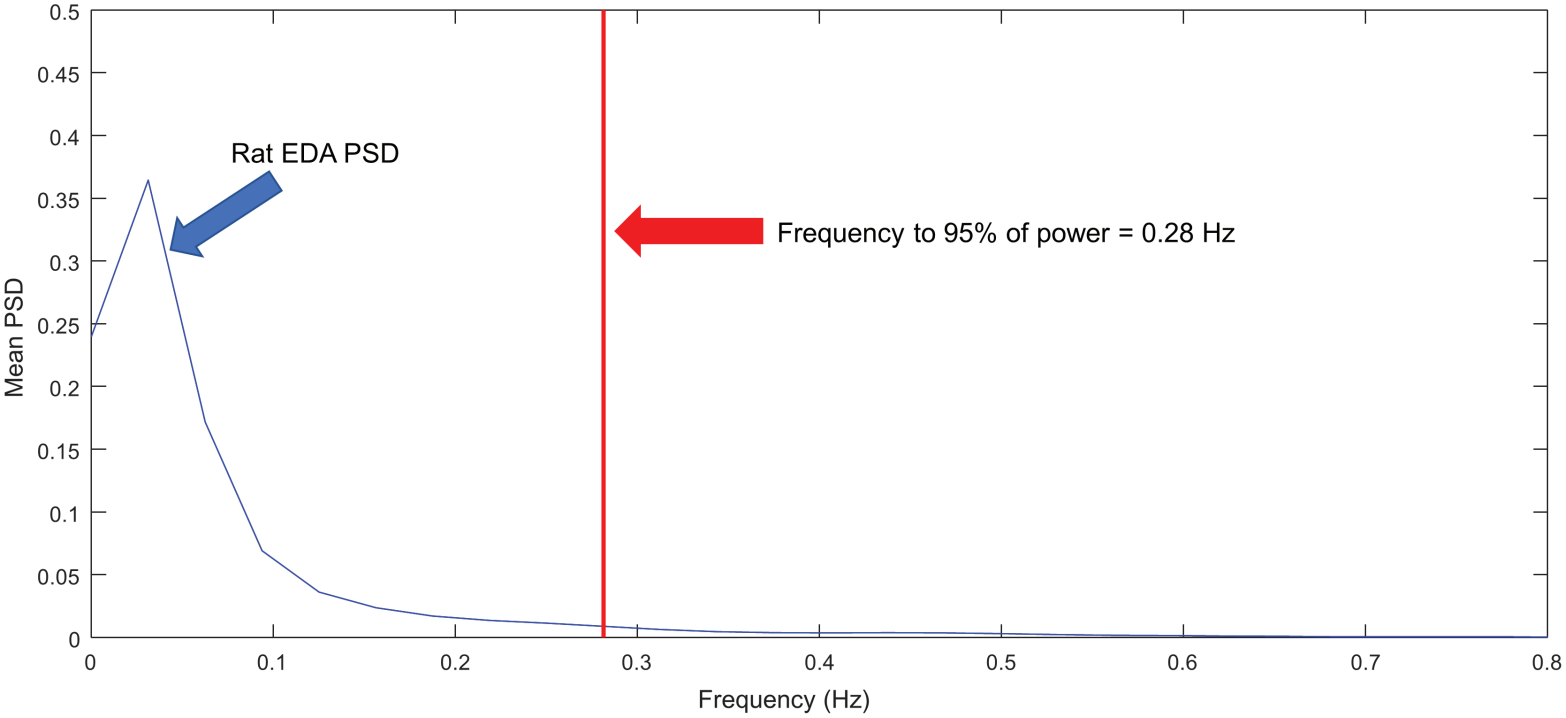
Ensemble of normalized power spectral densities of EDA signals for *N* = 10 rats.

Figure 9 shows the ensemble power spectral density (PSD) for the *N* = 10 rats included in the study. Most of the power is confined to frequencies below 0.1 Hz. The frequency that contains the 95% of the power is 0.28 Hz, which is roughly two times the frequency for humans at rest (Posada-Quintero et al., 2016a). The range of frequencies under stress and physical activity in humans can reach about 0.37 Hz. Furthermore, as observed in Figure 4, rat SCRs are faster than human SCRs. Based on this information, we have modified TVSymp, the second and third components, comprising the approximate range 0.16–0.48 Hz, as described in the methods section. This range of frequencies allowed us to incorporate the dynamics of EDA to capture the effects of CNS-OT in rats.

**Figure 10:**
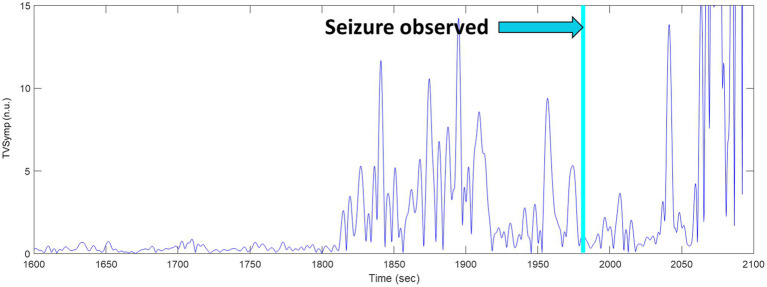
TVSymp elevation before seizure observed in a sample rat.

We observed an elevation in the TVSymp modified specifically for EDA signals obtained from rats. Figure 10 includes a closer look to the TVSymp for a given rat in the time preceding and following the seizure. For this specific rat, the elevation on TVSymp occurred about 1840 s after the chamber was pressurized. The seizure was observed about 140 s later (1980 s) in the plot.

**Figure 11:**
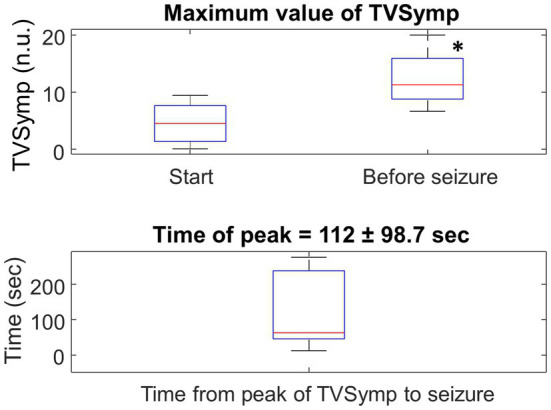
Top: Boxplot for the maximum value of TVSymp at the Start (chamber pressurized) and Before seizure for the entire population. *N* = 10, ^*^ represents significance difference to Start of HBO_2_ (*p* = 0.005). Bottom: Boxplot of the time-lapse from the peak of TVSymp to the occurrence of the seizure.

Figure 11 shows box plots for the maximum value of TVSymp at the Start and Before seizure for the *N* = 10 rats. There was a significant increase (two- to three-fold) in the maximum value of TVSymp between the two stages. The time of occurrence of the maximum value of TVSymp before seizure is 112 s (standard deviation = 98.7 s).

## Discussion

We have developed methods for measuring EDA in tethered, freely behaving male, Sprague Dawley rat during compression and exposure to HBO_2_ to test the hypothesis that dynamic changes in EDA, which are quantified as TVSymp (Posada-Quintero et al., 2016b), precede onset of seizures. Our findings support this hypothesis and reveal that changes in TVSymp activity begin ~2 min, on average, before onset of generalized seizures when rats breathe HBO_2_. Thus, increased dynamics in TVSymp activity are another “physio-marker” of an impending seizure during protracted exposure to HBO_2_ in rat and, presumably, other mammals, including humans. It will be important to determine how these findings translate to humans, especially under conditions of immersion which accelerates onset of CNS-OT (Ciarlone et al., 2019). Currently, the U. S. Navy’s guideline for time spent at 50 feet of seawater breathing pure O_2_ via a rebreather is 10 min (USN, 2016). Thus, an early physio-marker of ~2 min – 20% of the stated guideline – is a significant warning signal and provides sufficient to ascend partially to less depth, thus lowering the level of inspired PO_2_. Additionally, because of this physio-marker, and other potential mitigation strategies that extend bottom time (Stavitzski et al., 2021), if the diver does remain at 50 feet of seawater beyond 10 min, 2 min is still sufficient time to safely reduce their depth and even surface if the latter is possible. The lack of nitrogen in the breathing gas reduces the diver’s inert gas load and their risk for DCS during ascent. In addition, there are other physio-markers including early changes in cardio-respiration that precede seizure (Pilla et al., 2013b), which when combined with changes in EDA and TVSymp could be a useful multidimensional early warning set of physio-markers to incorporate into a monitoring device.

The Sprague Dawley rat has been widely used to study the early physiology that precedes seizure genesis during exposure to HBO_2_ in mammals; for example, rats begin to hyperventilate several minutes prior to seizure genesis, indicating hyperoxic hyperpnea/hyperventilation is a physio-marker of an impending seizure in rats (Pilla et al., 2013b). Humans, likewise, exhibit hyperoxic hyperventilation during prolonged exposure to normobaric hyperoxia^1^ and hyperbaric hyperoxia (Dean et al., 2004). In addition, Sprague Dawley rats have been used to test various drug therapies that may increase the risk of CNS-OT (Pilla et al., 2013a; Held et al., 2014) or, alternatively, provide neuroprotection against CNS-OT and delay onset of seizures (D’Agostino et al., 2013; Ari et al., 2019; Stavitzski et al., 2021). As in these previous animal studies, 5 ATA O_2_ was used here to accelerate onset of seizures in unanesthetized rats to avert the confounding problem of pulmonary oxygen toxicity, which takes longer to develop when breathing hyperoxia (Ciarlone et al., 2019). Our findings show that as in previous studies (D’Agostino et al., 2013; Pilla et al., 2013a,b; Held et al., 2014; Stavitzski et al., 2021), the duration of the safe latent period prior to seizure genesis is variable between unanesthetized rats, ranging from ~10–35 min. Regardless, the latency time to seizure, TVSymp activity increased on average, ~2 min prior to seizure.

Physiologically, increased sympathetic activity associated with augmented seizure activity occurs at 1 ATA when breathing air (Wannamaker, 1985). In humans, increased EDA results from the changes in conductance in the skin that are typically proportional to sweat secretion (Boucsein et al., 2012). In rodents, however, sweat glands are mostly located in palmar and plantar surfaces (Ring and Randall, 1947). Regardless, this study and others (Kita et al., 1975; Hata et al., 1981) report that good EDA signals can be obtained from subdermal electrodes placed on the rat’s back. Rodents exposed to HBO_2_ display enhanced autonomic neural activity that is measured as transient bradycardia followed by tachycardia, hypertension, increased cerebral blood flow, and hyperventilation; reviewed in (Ciarlone et al., 2019). Likewise, core body temperature often decreases, presumably due to increased tail skin blood flow and thus heat loss (Puglia et al., 1974; Fenton and Robinson, 1993). Raw EDA is a purely sympathetic assay (Dawson et al., 2007; Boucsein et al., 2012) and EDA has been used increasingly to assess the sympathetic activity (Freeman and Chapleau, 2013). Thus, given the significant amount of sympathetic perturbations that transpire during exposure to HBO_2_, and that precede seizure genesis, it is not surprising that the temporal pattern of EDA changed in the rat prior to seizures.

We have observed for the first time the spectral distribution of EDA signals and the characteristics of the SCRs in rats. In the spectral domain, we found that 95% of the power of EDA in rats is confined to frequencies below 0.28 Hz, at rest (Figure 4). That is approximately twice the frequency for humans, which is about 0.15 Hz (Posada-Quintero et al., 2016a). In humans, this frequency increases to 0.25 Hz under cognitive, orthostatic, and physical stress, and to about 0.37 Hz under vigorous physical activity (Posada-Quintero et al., 2018b). Further research is required to explore the spectral distribution of EDA in rats under stress or physical activity. Furthermore, the human and rat SCRs shown in Figure 4 show clear differences. In humans, rise time (the time from the onset of the SCR to the peak) ranges from 0.5 to 5 s. The human SCR shown in Figure 4 has a rise time of about 4 s, whereas the rise time for the rat SCR is about 0.7 s. We observed that rise time in rats did not exceed 1 s.

There are no signal processing techniques specifically developed for rats’ EDA (tonic/phasic decomposition), despite the recent advances of data collection and processing for EDA in humans (Posada-Quintero and Chon, 2020). Furthermore, the method for automatic assessment of EDA data quality, described as “simple, transparent, and flexible” was developed specifically for humans’ EDA (Kleckner et al., 2018). We modified the method and detected some instances of motion artifacts, but in other instances the method was not able to properly identify data corruption. To identify motion artifacts in rats’ EDA, a more detailed understanding of the signal is required. We used the video recordings to make sure the changes in EDA were not produced by motion.

For ethical purposes, it is impractical to induce seizures in human subjects. The animal model, however, enables the investigator to go further and measure the time between the elevation of EDA, and thus TVSymp, and seizures. If our ability to recognize early signs of CNS-OT holds up during further testing in humans, we will need to overcome the challenge to quantify the elevation in TVSymp to trigger a warning of seizures. Although a threshold value can be determined, this value is tuned from the collected dataset. We foresee the suitability of machine learning algorithms to automatically detect the risk of seizures development from TVSymp time series.

## Conclusion

We found that while the latency time to seizure is variable between animals, TVSymp is significantly increased about two minutes on average prior to seizures caused by breathing HBO_2_ in rats. The animal model used in this study suggests that TVSymp has the potential to enable the prediction of seizures caused during CNS-OT using a wearable sensor in humans, as EDA can be easily collected from several body sites. This is a promising finding, but further research is necessary to confirm the suitability of this tool to predict CNS-OT in humans.

## Data Availability Statement

The raw data supporting the conclusions of this article will be made available by the authors, without undue reservation.

## Ethics Statement

The animal study was reviewed and approved by the University of South Florida Institutional Animal Care and Use Committee (PHS assurance no. A4100-01; AAALACi 434) and the Department of the Navy, Bureau of Medicine and Surgery.

## Author Contributions

JD, CL, and NS designed and conducted the experiment and edited the manuscript and expanded the results. HP-Q and KC performed the analysis of data and wrote the first draft of the manuscript. All authors contributed to manuscript revision and read and approved the submitted version.

## Funding

This work was funded by ONR N00014-19-1-2314 (JD) and N00014-19-1-2209 (KC and HP-Q).

## Conflict of Interest

The authors declare that the research was conducted in the absence of any commercial or financial relationships that could be construed as a potential conflict of interest.

## Publisher’s Note

All claims expressed in this article are solely those of the authors and do not necessarily represent those of their affiliated organizations, or those of the publisher, the editors and the reviewers. Any product that may be evaluated in this article, or claim that may be made by its manufacturer, is not guaranteed or endorsed by the publisher.

## Footnotes

^1^Exposure to normobaric hyperoxia initially inhibits respiration by carotid body “chemodenervation.” The inhibitory effect of hyperoxia, however, is only transient and continued exposure causes a central stimulation after several minutes that is significant especially if isocapnic hyperoxia is used (Dean et al., 2004).

## References

[ref1] Ari C. Koutnik A. P. DeBlasi J. Landon C. Rogers C. Q. Vallas J.. (2019). Delaying latency to hyperbaric oxygen-induced CNS oxygen toxicity seizures by combinations of exogenous ketone supplements. Phys. Rep. 7:e13961. doi: 10.14814/phy2.13961, PMID: 30604923 PMC6317287

[ref2] Arieli R. Shochat T. Adir Y. (2006). CNS Toxicity in Closed-Circuit Oxygen Diving: Symptoms Reported from 2527 Dives. Aviat. Space Environ. Med. 77, 526–532. PMID: 16708533

[ref3] Bai Y. (2011). Evaluation of the Effects of Hyperbaric Dive Environments on the Autonomic Nervous System Using Principal Dynamic Mode Analysis. Am. J. Physiol. Regul. Integr. Comp. Physiol. 305, R748–R758. doi: 10.1152/ajpregu.00150.2012 23883677

[ref4] Blogg S. L. Gennser M. Loveman G. A. M. Seddon F. M. Thacker J. C. White M. G.. (2003). The effect of breathing hyperoxic gas during simulated submarine escape on venous gas emboli and decompression illness. Undersea Hyperbaric Med. Bethesda 30, 163–174. 14620096

[ref5] Boucsein W. Fowles D. C. Grimnes S. Ben-Shakhar G. Roth W. T. Dawson M. E.. (2012). Publication recommendations for electrodermal measurements. Psychophysiology 49, 1017–1034. doi: 10.1111/j.1469-8986.2012.01384.x, PMID: 22680988

[ref6] Chon K. H. Dash S. Ju K. (2009). Estimation of respiratory rate from photoplethysmogram data using time-frequency spectral estimation. I.E.E.E. Trans. Biomed. Eng. 56, 2054–2063. doi: 10.1109/TBME.2009.2019766, PMID: 19369147

[ref7] Chouchou F. Pichot V. Garet M. Barthélémy J.-C. Roche F. (2009). Dominance in cardiac parasympathetic activity during real recreational SCUBA diving. Eur. J. Appl. Physiol. 106, 345–352. doi: 10.1007/s00421-009-1010-0, PMID: 19277697

[ref8] Ciarlone G. E. Hinojo C. M. Stavitzski N. M. Dean J. B. (2019). CNS function and dysfunction during exposure to hyperbaric oxygen in operational and clinical settings. Redox Biol. 27:101159. doi: 10.1016/j.redox.2019.101159, PMID: 30902504 PMC6859559

[ref9] Clark J. M. Neuman T. S. (2008). Physiology and Medicine of Hyperbaric Oxygen Therapy.

[ref11] Curly M. D. Ryder S. Harabin A. (1997). Medical Preparedness for Submarine Escape and Rescue. Medical Preparedness for Submarine Escape and Rescue. Groton, CT: Naval Submarine Medical Research Laboratory.

[ref12] D’Agostino D. P. Pilla R. Held H. E. Landon C. S. Puchowicz M. Brunengraber H.. (2013). Therapeutic ketosis with ketone ester delays central nervous system oxygen toxicity seizures in rats. Am. J. Phys. Regul. Integr. Comp. Phys. 304, R829–R836. doi: 10.1152/ajpregu.00506.2012, PMID: 23552496

[ref13] Dainer H. Nelson J. Brass K. Montcalm-Smith E. Mahon R. (2007). Short oxygen prebreathing and intravenous perfluorocarbon emulsion reduces morbidity and mortality in a swine saturation model of decompression sickness. J. Appl. Physiol. 102, 1099–1104. doi: 10.1152/japplphysiol.01539.2005, PMID: 17095628

[ref14] Dawson M. E. Schell A. M. Filion D. L. (2007). “The electrodermal system,” in Handbook of psychophysiology. eds. Cacioppo John T. Tassinary Louis G. Berntson Gary (United Kingdom: Cambridge university press), 159–181.

[ref15] Dean J. B. Mulkey D. K. Garcia A. J. Putnam R. W. Henderson R. A. (2003). Neuronal sensitivity to hyperoxia, hypercapnia, and inert gases at hyperbaric pressures. J. Appl. Physiol. 1985, 883–909. doi: 10.1152/japplphysiol.00920.2002 12909594

[ref16] Dean J. B. Mulkey D. K. Henderson R. A. Potter S. J. Putnam R. W. (2004). Hyperoxia, reactive oxygen species, and hyperventilation: oxygen sensitivity of brain stem neurons. J. Appl. Physiol. 1985, 784–791. doi: 10.1152/japplphysiol.00892.2003 14715688

[ref17] Demchenko I. T. Boso A. E. O’Neill T. J. Bennett P. B. Piantadosi C. A. (2000). Nitric oxide and cerebral blood flow responses to hyperbaric oxygen. J. Appl. Physiol. 1985, 1381–1389. doi: 10.1152/jappl.2000.88.4.1381 10749833

[ref18] Demchenko I. T. Welty-Wolf K. E. Allen B. W. Piantadosi C. A. (2007). Similar but not the same: normobaric and hyperbaric pulmonary oxygen toxicity, the role of nitric oxide. Am. J. Phys. Lung Cell. Mol. Phys. 293, L229–L238. doi: 10.1152/ajplung.00450.2006, PMID: 17416738

[ref19] Donald K. W. (1947). Oxygen poisoning in man. Br. Med. J. 1:712. 20248096 10.1136/bmj.1.4507.712 PMC2053400

[ref20] Donald K. (1992). Oxygen and the Diver. Best Pub Co. Available at: https://www.amazon.com/Oxygen-Diver-Kenneth-Donald/dp/1854211765

[ref21] Fenton L. H. Robinson M. B. (1993). Repeated exposure to hyperbaric oxygen sensitizes rats to oxygen-induced seizures. Brain Res. 632, 143–149. doi: 10.1016/0006-8993(93)91149-M, PMID: 8149223

[ref22] Freeman R. Chapleau M. W. (2013). Testing the autonomic nervous system. Handb. Clin. Neurol. 115, 115–136. doi: 10.1016/B978-0-444-52902-2.00007-2 23931777

[ref23] Gehrmann J. Hammer P. E. Maguire C. T. Wakimoto H. Triedman J. K. Berul C. I. (2000). Phenotypic screening for heart rate variability in the mouse. Am. J. Physiol. Heart Circ. Physiol. 279, H733–H740. doi: 10.1152/ajpheart.2000.279.2.H733, PMID: 10924073

[ref24] Gempp E. Louge P. (2013). Inner ear decompression sickness in scuba divers: a review of 115 cases. Eur. Arch. Otorhinolaryngol. 270, 1831–1837. doi: 10.1007/s00405-012-2233-y, PMID: 23100085

[ref25] Gempp E. Louge P. Blatteau J. E. Hugon M. (2012). Risks factors for recurrent neurological decompression sickness in recreational divers: a case-control study. J. Sports Med. Phys. Fitness 52, 530–536. PMID: 22976740

[ref26] Hampson N. Atik D. (2003). Central nervous system oxygen toxicity during routine hyperbaric oxygen therapy. Undersea Hyperb. Med. 30, 147–153. PMID: 12964858

[ref27] Hata T. Kita T. Yoneda R. Tanada S. (1981). Effects of Exogenous Stimuli and Centrally Acting Drugs on Galvanic Skin Responses in Rats. Jpn. J. Pharmacol. 31, 23–31. doi: 10.1254/jjp.31.23 7253342

[ref28] Held H. E. Pilla R. Ciarlone G. E. Landon C. S. Dean J. B. (2014). Female rats are more susceptible to central nervous system oxygen toxicity than male rats. Phys. Rep. 2:e00282. doi: 10.14814/phy2.282, PMID: 24771690 PMC4001875

[ref29] Huang N. E. Shen Z. Long S. R. Wu M. C. Shih H. H. Zheng Q.. (1998). The empirical mode decomposition and the Hilbert spectrum for nonlinear and non-stationary time series analysis. Proc. Royal Soc. London A 454, 903–995. doi: 10.1098/rspa.1998.0193

[ref30] Kita T. Hata T. Yoneda R. Okage T. (1975). Stress state caused by alteration of rhythms in environmental temperature, and the functional changes in mice and rats. Nihon Yakurigaku Zasshi 71, 195–210. 1169194 10.1254/fpj.71.195

[ref31] Kleckner I. R. Jones R. M. Wilder-Smith O. Wormwood J. B. Akcakaya M. Quigley K. S.. (2018). Simple, Transparent, and Flexible Automated Quality Assessment Procedures for Ambulatory Electrodermal Activity Data. I.E.E.E. Trans. Biomed. Eng. 65, 1460–1467. doi: 10.1109/TBME.2017.2758643, PMID: 28976309 PMC5880745

[ref32] Lund V. Kentala E. Scheinin H. Klossner J. Sariola-Heinonen K. Jalonen J. (2000). Hyperbaric oxygen increases parasympathetic activity in professional divers. Acta Physiol. Scand. 170, 39–44. doi: 10.1046/j.1365-201x.2000.00761.x, PMID: 10971221

[ref33] Massey F. J. Jr. (1951). The Kolmogorov-Smirnov test for goodness of fit. J. Am. Stat. Assoc. 46, 68–78. doi: 10.1080/01621459.1951.10500769, PMID: 34538265

[ref34] Miller L. H. (1956). Table of percentage points of Kolmogorov statistics. J. Am. Stat. Assoc. 51, 111–121. doi: 10.1080/01621459.1956.10501314

[ref35] Moon R. E. (2014). Hyperbaric oxygen treatment for decompression sickness. Undersea Hyperb. Med. 41, 151–157. PMID: 24851553

[ref36] Natoli M. J. Vann R. D. (1996). Factors Affecting CNS Oxygen Toxicity in Humans. Duke univ medical center durham nc fg hall lab for environmental research.

[ref37] Pilla R. Held H. E. Landon C. S. Dean J. B. (2013a). High doses of pseudoephedrine hydrochloride accelerate onset of CNS oxygen toxicity seizures in unanesthetized rats. Neuroscience 246, 391–396. doi: 10.1016/j.neuroscience.2013.04.035, PMID: 23624060

[ref38] Pilla R. Landon C. S. Dean J. B. (2013b). A potential early physiological marker for CNS oxygen toxicity: hyperoxic hyperpnea precedes seizure in unanesthetized rats breathing hyperbaric oxygen. J. Appl. Physiol. 114, 1009–1020. doi: 10.1152/japplphysiol.01326.2012 23429869

[ref39] Poh M.-Z. Loddenkemper T. Reinsberger C. Swenson N. C. Goyal S. Sabtala M. C.. (2012). Convulsive seizure detection using a wrist-worn electrodermal activity and accelerometry biosensor. Epilepsia 53:444. doi: 10.1111/j.1528-1167.2012.03444.x, PMID: 22432935

[ref40] Poh M.-Z. Loddenkemper T. Swenson N. C. Goyal S. Madsen J. R. Picard R. W. (2010). Continuous monitoring of electrodermal activity during epileptic seizures using a wearable sensor. in Engineering in Medicine and Biology Society (EMBC), 2010 Annual International Conference of the IEEE (IEEE), 4415–4418. 10.1109/IEMBS.2010.5625988 21095760

[ref41] Posada-Quintero H. F. Bolkhovsky J. B. Reljin N. Chon K. H. (2017). Sleep Deprivation in Young and Healthy Subjects Is More Sensitively Identified by Higher Frequencies of Electrodermal Activity than by Skin Conductance Level Evaluated in the Time Domain. Front. Physiol. 8, 1–9. doi: 10.3389/fphys.2017.00409 28676763 PMC5476732

[ref42] Posada-Quintero H. F. Chon K. H. (2020). Innovations in Electrodermal Activity Data Collection and Signal Processing: A Systematic Review. Sensors 20:479. doi: 10.3390/s20020479, PMID: 31952141 PMC7014446

[ref43] Posada-Quintero H. F. Dimitrov T. Moutran A. Park S. Chon K. H. (2019). Analysis of Reproducibility of Noninvasive Measures of Sympathetic Autonomic Control Based on Electrodermal Activity and Heart Rate Variability. IEEE Access 7, 22523–22531. doi: 10.1109/ACCESS.2019.2899485

[ref44] Posada-Quintero H. F. Florian J. P. Orjuela-Cañón A. D. Aljama-Corrales T. Charleston-Villalobos S. Chon K. H. (2016a). Power Spectral Density Analysis of Electrodermal Activity for Sympathetic Function Assessment. Ann. Biomed. Eng. 44, 3124–3135. doi: 10.1007/s10439-016-1606-6 27059225

[ref45] Posada-Quintero H. F. Florian J. P. Orjuela-Cañón Á. D. Chon K. H. (2016b). Highly sensitive index of sympathetic activity based on time-frequency spectral analysis of electrodermal activity. Am. J. Phys. Regul. Integr. Comp. Phys. 311, R582–R591. doi: 10.1152/ajpregu.00180.2016 27440716

[ref46] Posada-Quintero H. F. Florian J. P. Orjuela-Cañón A. D. Chon K. H. (2018a). Electrodermal Activity Is Sensitive to Cognitive Stress under Water. Front. Physiol. 8:1128. doi: 10.3389/fphys.2017.01128 29387015 PMC5776121

[ref47] Posada-Quintero H. F. Kong Y. Nguyen K. Tran C. Beardslee L. Chen L.. (2020a). Using electrodermal activity to validate multilevel pain stimulation in healthy volunteers evoked by thermal grills. Am. J. Phys. Regul. Integr. Comp. Phys. 319, R366–R375. doi: 10.1152/ajpregu.00102.2020 PMC7509251 32726157

[ref48] Posada-Quintero H. F. Reljin N. Mills C. Mills I. Florian J. P. VanHeest J. L.. (2018b). Time-varying analysis of electrodermal activity during exercise. PLoS One 13:e0198328. doi: 10.1371/journal.pone.0198328 29856815 PMC5983430

[ref49] Posada-Quintero H. F. Reljin N. Moutran A. Georgopalis D. Lee E. C.-H. Giersch G. E. W.. (2020b). Mild Dehydration Identification Using Machine Learning to Assess Autonomic Responses to Cognitive Stress. Nutrients 12:42. doi: 10.3390/nu12010042 PMC7019291 31877912

[ref50] Puglia C. D. Glauser E. M. Glauser S. C. (1974). Core temperature response of rats during exposure to oxygen at high pressure. J. Appl. Physiol. 36, 149–153. doi: 10.1152/jappl.1974.36.2.149, PMID: 4811373

[ref51] Ring J. R. Randall W. C. (1947). The distribution and histological structure of sweat glands in the albino rat and their response to prolonged nervous stimulation. Anat. Rec. 99, 7–19. doi: 10.1002/ar.1090990103, PMID: 20262457

[ref52] Schipke J. D. Pelzer M. (2001). Effect of immersion, submersion, and scuba diving on heart rate variability. Br. J. Sports Med. 35, 174–180. doi: 10.1136/bjsm.35.3.174, PMID: 11375876 PMC1724326

[ref53] Simon A. J. Torbati D. (1982). Effects of hyperbaric oxygen on heart, brain, and lung functions in rat. Undersea Biomed. Res. 9, 263–275 PMID: 7135636

[ref54] Stavitzski N. M. Landon C. S. Hinojo C. M. Poff A. M. Rogers C. Q. D’Agostino D. P.. (2021). Exogenous ketone ester delays CNS oxygen toxicity without impairing cognitive and motor performance in male Sprague-Dawley rats. Am. J. Phys. Regul. Integr. Comp. Phys. 321, R100–R111. doi: 10.1152/ajpregu.00088.2021, PMID: 34132115

[ref10] USN. (2016). SS521-AG-PRO-010 US DIVING MANUAL REVISION 7 Volumes 1–5 Complete. Direction of commander, Naval sea systems command.

[ref55] Wang J. Tsang W. W. Marsaglia G. (2003). Evaluating Kolmogorov’s distribution. J. Stat. Softw. 8, 1–4.

[ref56] Wannamaker B. B. (1985). Autonomic Nervous System and Epilepsy. Epilepsia 26, S31–S39. doi: 10.1111/j.1528-1157.1985.tb05722.x, PMID: 3888616

[ref57] Weathersby P. K. Survanshi S. S. Parker E. C. Temple D. J. Toner C. B. (1999). Estimated DCS Risks in Pressurized Submarine Rescue. Naval Medical Research Inst Bethesda Md.

